# Virus-Heat Shock Protein Interaction and a Novel Axis for Innate Antiviral Immunity

**DOI:** 10.3390/cells1030646

**Published:** 2012-09-11

**Authors:** Mi Young Kim, Michael Oglesbee

**Affiliations:** Department of Veterinary Biosciences, The Ohio State University, 1925 Coffey Road, Columbus, OH 43210, USA; Email: Michael.Oglesbee@cvm.osu.edu

**Keywords:** 70 kDa heat shock protein (hsp70), innate immunity, interferon, virus, measles virus

## Abstract

Virus infections induce heat shock proteins that in turn enhance virus gene expression, a phenomenon that is particularly well characterized for the major inducible 70 kDa heat shock protein (hsp70). However, hsp70 is also readily induced by fever, a phylogenetically conserved response to microbial infections, and when released from cells, hsp70 can stimulate innate immune responses through toll like receptors 2 and 4 (TLR2 and 4). This review examines how the virus-hsp70 relationship can lead to host protective innate antiviral immunity, and the importance of hsp70 dependent stimulation of virus gene expression in this host response. Beginning with the well-characterized measles virus-hsp70 relationship and the mouse model of neuronal infection in brain, we examine data indicating that the innate immune response is not driven by intracellular sensors of pathogen associated molecular patterns, but rather by extracellular ligands signaling through TLR2 and 4. Specifically, we address the relationship between virus gene expression, extracellular release of hsp70 (as a damage associated molecular pattern), and hsp70-mediated induction of antigen presentation and type 1 interferons in uninfected macrophages as a novel axis of antiviral immunity. New data are discussed that examines the more broad relevance of this protective mechanism using vesicular stomatitis virus, and a review of the literature is presented that supports the probable relevance to both RNA and DNA viruses and for infections both within and outside of the central nervous system.

## 1. Introduction

Cellular heat shock proteins (HSPs) are one of the most phylogenetically conserved classes of proteins with critical roles in maintaining cellular homeostasis and in protecting the cells from stressful conditions, reflecting in large part their ability to facilitate folding of nascent protein or refolding of denatured protein [1]. HSPs are present in a wide variety of organisms, tissues, and cells, and occupy diverse intracellular locations including the cytosol of prokaryotes, and the cytosol, nucleus, endoplasmic reticulum, mitochondria and chloroplasts of eukaryotes [2]. HSPs are classified into families according to their mass. These include small HSP, HSP40, HSP60, HSP70, HSP90, and HSP110. Each HSP family contains proteins with closely related structures, whereas HSP families show little obvious amino acid homology to one another [3].

Since their initial discovery, diverse functions of HSPs have been elucidated. As molecular chaperones, HSPs maintain protein conformation, facilitate trafficking and assembly/disassembly of multimeric complexes [1]. But HSP may also bind native protein, altering their conformation and thus function. This type of interaction is known as HSP-mediated activity control and is in contrast to the stability control associated with HSP-mediated protein folding events [4].

While HSPs are host protective in times of cell stress, they also support gene expression of DNA and RNA viruses, particularly for viruses interacting with the major inducible 70kDa HSP isoform (hsp70). Hsp70 is present in all organisms, ranging from archaebacteria and plants to humans [2,5]. Expression levels of hsp70 vary, with hsp70 being induced and redistributed within cells in support of virus replication and being induced in tissues by febrile temperatures that frequently accompany viral infection. As such, levels of hsp70 may determine infection phenotype through direct support of viral gene expression. Hsp70 also plays an immune modulatory role, highlighting potential to influence innate and adaptive immune response against viral infection. The focus of this review will therefore examine the potential of virus-hsp70 interaction to influence outcome of infection, taking into consideration the role of hsp70 in virus replication as well as innate immune responses. Particular emphasis will be placed on infections of the central nervous system, highlighting animal models that have provided the first insights into the biological significance of virus-hsp70 interaction.

## 2. The 70 kDa Heat Shock Protein Family (HSP70)

Members of the 70 kDa HSPs family (HSP70) are highly conserved across species. The human HSP70 family includes at least eight unique proteins that differ from each other by level of expression and cellular localization. Hsp70-5(Grp78) and hsp70-9(Grp75) are located in the lumen of the endoplasmic reticulum and in the mitochondrial matrix, respectively. The remaining six HSP70 proteins are hsp70-1a, hsp70-1b, hsp70-1t, hsp70-2, hsp70-6, and hsc70, all of which reside in the cytosol and nucleus. Cytosolic HSP70 family members include a constitutively expressed isoform, hsc70 (or hsc73) and two major stress-inducible members, hsp70-1a and 1b that differ by only two amino acids and are collectively known as hsp70 (also referred to hsp72 in order to facilitate distinction from the family as a whole). For both humans and mice, hsp70-1a and hsp70-1b are encoded by two closely linked, intronless stress-inducible genes (HSPA1A and HSPA1B in humans) located in the major histocompatibility (MHC) class III cluster [6]. There is 50% amino acid identity between human hsp70 and the prokaryotic homologue DnaK, and 76% identity between human hsp70 and yeast SSA. The conservation of hsp70 sequence reflects similar functional properties across species. Rodent hsp70 can be functionally complimented by human hsp70 to induce cellular protection against various stress responses [7,8]. 

Overexpression of hsp70 increases resistance and protection against heat, ischemia, and oxidative stress [9,10,11,12,13]. Protection is attributed to the chaperone functions of hsp70 that support protein folding, translocation, and assembly reactions [14,15,16,17] and prevention of denatured protein aggregation [1,18]. These same functions would support maturation of high levels of viral protein typical of lytic infections. Hsp70 activity control functions include inhibition of apoptosis. Hsp70 inhibits the signal transduction pathways of apoptosis, including Bax translocation into mitochondria, release of cytochrome c from mitochondria, apoptosome assembly through interactions with Apaf-1, and activation of initiator caspase 3 [19]. Accordingly, inhibition of hsp70 expression in human breast tumor cells result in massive cell death [20]. Inhibition of apoptosis may enhance viral replication by preserving the viral factory. Conversely, adenovirus infection causes a dramatic suppression of hsp70 mRNA levels during the late phases of the viral replication cycle when the viral particles are already assembled, enhancing apoptosis in order to promote viral particle release [21].

### 2.1. Viral Induction of hsp70 (the Major Inducible HSP70 Family Member)

Heat shock factor 1 (HSF-1) is the primary transcription factor involved in the induction of hsp70 (*i.e.*, hsp70-1a). Both hsc70 and hsp70 bind the inactive monomeric form of HSF-1 during cellular homeostasis [22]. In response to changes in cellular environment, the HSPs are titrated away from HSF-1 to new client proteins, allowing HSF-1 trimerization and phosphorylation activation [23]. The active phosphorylated HSF-1 then migrates to the nucleus to increase HSP transcription by binding heat shock responsive elements in the promoter [24]. Promoter elements may also respond to other transcription factors that include transactivating viral proteins [25]. 

Expression levels of hsp70 vary widely, being expressed at varying constitutive levels as a function of tissue and animal species, and being highly induced by physiological stimuli such as fever [26]. Induction of hsp70 by febrile temperatures involves multiple tissues, including brain [27]. Virus infection induces hsp70 expression both directly [28] and indirectly as a consequence of the febrile response that frequently accompanies virus infection [29]. For example, fever is a consistent sequel to measles virus (MeV) infection [30], being used as a diagnostic criterion in clinical settings, with temperature elevations as low as 39 °C being a potent inducer of hsp70 in the human brain [27]. Virus-induced inflammatory cytokines may also represent a means of indirect hsp70 induction. The latter is supported by findings in canine distemper virus infected dog brains, where hsp70 induction was observed in viral antigen negative as well as viral antigen positive astrocytes, these being restricted to areas of active viral replication and inflammation [29]. Direct virus induction of hsp70 may reflect an unfolded protein response. Here, hsp70 induction follows the production of a large number of proteins requiring chaperones to mediate proper folding and / or assembly, or the induction of protein misfolding as cellular homeostasis is lost (e.g., progression of virus-induced cytopathic effect). Nascent viral protein or misfolded cellular proteins compete with heat shock factor(HSF) for hsp70 binding, resulting in increased levels of free and active HSF [31]. This unfolded protein response has been shown in several virus infections, particularly well-illustrated for plant viruses. Tobacco mosaic virus induction of hsp70 parallels the concentration of the insoluble viral coat protein, supporting the view that viral protein is a potent inducer of chaperones including hsp70 [32]. Studies with turnip mosaic virus and turnip crinkle virus show that hsp70 induction may not be specific to the expression of any one viral structural protein, but rather due the accumulation of viral protein in general [33]. Unfolded protein responses may involve more than just hsp70. Within the endoplasmic reticulum, Grp78/Bip (Hsp70-5) and Grp94 (Hsp90) are induced during Hepatitis C virus infection by interaction with viral envelope protein E2 protein, where the chaperones retain misfolded viral proteins in a pre-Golgi compartment [34].

Direct viral induction of hsp70 may also reflect a more highly selective (specific) process. Selective hsp70 induction was demonstrated in adenovirus type5 (Ad5), simian virus (SV40) and human cytomegalovirus (HCMV) infection. Here, transactivating viral proteins interact directly with promoter-specific transcription factors or with cis-acting DNA elements. The Ad5 early gene product E1A selectively stimulates hsp70 gene transcription through interaction with the CCAAT-Box binding factor [35].The E1B gene product also mediates post transcriptional regulation of hsp70 expression, enhancing the export of hsp70 mRNA to the cytoplasm during late stages of infection [36]. The SV40 large T antigen induces hsp70 transcription by stabilizing the TATA binding protein (TBP) and TFIIA complex on hsp70 TATA promoter elements [37]. The immediate-early (IE) 2 protein of HCMV trans-activates the hsp70 promoter in a TATA box dependent manner by direct interaction with TFIID [38]. These more selective mechanisms of virus-induced hsp70 expression strongly suggest that hsp70 plays key roles in support of virus replication. 

### 2.2. The Role of the Major Inducible 70 kDa HSP (hsp70) in Support of Viral Replication

When looking at the HSP70 family as a whole, their fundamental role in protein metabolism and the abundance within cells reinforces their potential support of viral replication at multiple levels [39]. For every step in viral replication (*i.e.*, attachment/penetration, uncoating, transcription and genome replication, and virion morphogenesis), one can cite an example of a viral system that draws upon an HSP70 family member for support. Those roles are different for constitutively expressed isoforms(hsc70 and Grp78) and highly inducible isoforms(*i.e.*, hsp70) [28]. In general, hsc70 and Grp78 (Bip) support protein folding / unfolding and assembly / disassembly reactions required for viral uncoating, membrane glycoprotein maturation, and nucleocapsid assembly [40]. In contrast, hsp70 is directly involved in viral transcriptase and / or replicase activity. Mechanistically, this aspect of virus-hsp70 interaction is best understood for DNA viruses (reviewed [28], Table 1). Hsp70 stimulates transcription and replication of DNA virus including herpes simplex virus type 1(HSV-1) and human papillomavirus by mediating assembly of preinitiation complexes on the origin of DNA replication [41,42].

We have studied the role(s) of hsp70 in stimulating viral gene expression by measles virus (MeV) and canine distemper virus (CDV), negative strand RNA viruses in the family Paramyxoviridae. Selective overexpression of hsp70 through stable transfection or induction through transient heat shock increases viral transcription and replication following infection, resulting in elevated viral protein expression and cytopathic effect [43,44,45,46,47]. Hsp70-dependent gene expression reflects direct interaction between hsp70 and viral nucleocapsid which consists of single stranded negative sense genomic RNA packed by nucleocapsid protein (N) [46,47,48]. The nucleocapsid is the self-contained means of viral gene expression, possessing a viral RNA-dependent RNA polymerase (L) protein that functions as both transcriptase and replicase. The viral polymerase is tethered to the N protein-RNA genome template by the P protein; the RNA genome is not directly bound by L. Addition of purified hsp70 enhances nucleocapsid transcriptional activity in cell-free assays, and hsp70-specific antibody suppresses that activity. The mechanism by which hsp70 enhances transcription and genome replication is unclear at present, although models include hsp70-dependent changes in nucleocapsid morphology that is linked to template function, and direct modification of P–N binding [49]. Progression of the polymerase along the template should require cycles of P–N binding and release. The high affinity of P-N binding would thus be rate limiting to the progressive movement of the polymerase along the template. Hsp70 and P both recognize the same C-terminal N protein sequences, and hsp70 can competitively inhibit P–N binding. These results suggest a polymerase cofactor function for hsp70, in which hsp70 facilitates cycles of P–N binding and release [49,50].

**Table 1:** Virus-hsp70 interaction in support of viral replication.

Virus	Viral Target of Hsp70 Interaction	Effect of Virus-Hsp70 Interaction	Reference
human papillomavirus	E1, E2	increased genome replication	[42]
herpes simplex virus type 1	UL9	increased genome replication	[41]
canine distemper virus, measles virus	nucleocapsid	increased nucleocapsid transcriptional activity and genome replication	[43,44,45,46,47]
respiratory syncytial virus	nucleocapsid	increased nucleocapsid transcriptional activity	[51]
rabies virus	nucleocapsid	support formation of replication factories (Negri bodies)	[52]
tomato bush stunt virus	P33	support replicase function	[53,54]
simian virus 40	Tag	Genome replication	[55]
human cytomegalovirus	Hap46	stimulate viral transcriptional activity	[56]

Another function by which hsp70 may influence paramyxovirus transcription and replication is by mediating nucleocapsid protein trafficking to cytoplasmic lipid membrane rafts, sites for the assembly of transcriptases and replicases [51]. This mechanism is supported by studies with the paramyxovirus respiratory syncytial virus (RSV), where hsp70-N interaction results in co-localization to lipid rafts. Cell-free RSV nucleocapsid transcriptional activity is detected within the insoluble cell fraction containing these rafts, and suppressed by antibody against hsp70. The latter is identical to our results with CDV and suggests that hsp70 may also be an integral component of transcriptase, which could include the functions mentioned above (e.g., influence nucleocapsid morphology or progressive movement of the viral polymerase complex). A role for hsp70 in supporting assembly of polymerase complexes on lipid platforms may be a more general phenomenon for RNA viruses replicating in the cytoplasm. Rabies virus infection of neurons induce nucleocapsid complexes that form on membranes derived from late endosomes and the Golgi, and these complexes are composed of a lipid core, viral nucleocapsid, hsp70, and α-tublin [52]. Moreover, plant viruses also require hsp70 to support viral gene expression that is linked to intracellular membranes. It was shown that SSA(homologue of hsp70) support stabilization of replicase function for tomato bushy stunt virus by mediating its localization to peroxisomal membranes. This observation was based upon use of a yeast library of genomic deletions to study function of host proteins required for viral replication [53,54]. 

### 2.3. Hsp70 Levels and the Virus Infection Phenotype

Hsp70-dependent levels of viral transcription can result in dramatic effects on the *in vitro* infection phenotype of MeV and CDV [45,46,47,57]. Early studies showed that heat shock induction of hsp70 in mink lung cells or mouse neuroblastoma cells converted a stable persistent CDV infection to a lytic infection phenotype [45]. The poorly productive non-cytopathic infections were converted to one in which infectious viral progeny were increased, and cells formed large syncytia and cytoplasmic inclusion bodies, with the rapid induction of cell lysis. Heat shock induces multiple HSP family members. The specific role of hsp70 in altering virus infection phenotype was shown via stable transfection to selectively express hsp70. Constitutive overexpression of hsp70 in human astrocytes increases MeV gene expression and cytopathic effect, that latter being illustrated by the formation of large plaques [47]. Mouse neuroblastoma cells also support increased MeV transcript and genome levels when transfected to constitutively express human hsp70 [43]. Rodent cells are a particularly good system to examine the effects of hsp70 on virus gene expression since rodents, unlike primates, lack significant constitutive hsp70 expression [27,58]. As such, hsp70 elevations mediated by hyperthermic treatment or transfection are markedly contrasted against the lack of basal expression in control (untreated) cells. The relatively limited permissiveness of mouse cells to MeV replication, in contrast to the greater permissiveness of primate cells, has been attributed to differences in the basal levels of hsp70 in the infected cell [59]. Mouse cells that are stably transfected to constitutively express hsp70 may thus be considered more human-like in their support of MeV replication.

The mouse model of MeV brain infection established the *in vivo* significance of hsp70-mediated increases in MeV gene expression, where virus-hsp70 interaction within the infected cell resulted in increased neurovirulence. Mice exhibit an age and strain-dependent susceptibility to viral neurovirulence following intracranial inoculation [60]. Neonatal mice support neuronal infection, and mice of the H-2^b^ haplotype (e.g., C57BL/6) exhibit enhanced susceptibility to infection due to an impaired antiviral immune response. Transgenic (TG) mice were created to selectively and constitutively express hsp70 in neurons. Intracranial inoculation of MeV in neonatal TG mice resulted in significantly increased brain viral RNA burdens and virus induced cytopathic effects relative to NT mice, with cytopathic effects including formation of neuronal syncytia, intranuclear and cytoplasmic inclusion bodies, and neuronal death [61]. These results are consistent with hsp70-dependent increases in viral gene expression being the basis for increased neurovirulence, and this was proven by infecting mice with a genetically engineered virus lacking a transcriptional response to hsp70. Hsp70 did not influence the outcome of infection for virus lacking a transcriptional response to hsp70. These results indicate the potential of hsp70 to influence virus infection phenotype *in vitro* and *in vivo*, but the *in vivo* system was tested in a host that was deficient in virus-specific immune responses. What is the virus-hsp70 relationship in an immune competent host? In addressing this question, we will first review what is known about the potential for extracellular release of hsp70 that might occur as a consequence of virus infection, and the immune modulatory roles of extracellular hsp70.

## 3. Extracellular hsp70

Hsp70 can be released from cells following cell death [62], but also via active processes independent of cell death [63,64]. Release following lytic infection has been shown with parvovirus infection of a tumor cell line, where cell death released hsp70 in addition to other heat shock protein family members including hsp60 and hsp90 [65]. We have shown similar virus-induced release of hsp70 in mouse neuroblastoma cells lytically infected with MeV and vesicular stomatitis virus (VSV), where the concentration of intracellular hsp70 is proportionate to the level of hsp70 release (unpublished observation). VSV infection of neuroblastoma cells is highly cytopathic (*i.e.*, lytic). MeV infection of neuroblastoma cells is typically non-cytopathic, although we were able to create a lytic infection phenotype by transfecting cells to express a receptor used preferentially by the infecting strain of MeV, in this case the CD46 receptor and the Edmonston strain of MeV. Lytic infections of control neuroblastoma cells resulted viral induction of hsp70 and low level release. Release was dramatically increased in cells that were stably transfected to constitutively express hsp70.

We have also demonstrated hsp70 secretory release from non-cytopathic MeV infection of neuroblastoma cells (unpublished observation). Secretory release of hsp70 is well documented in exosomal pathways. Exosomes are 40 to 100 nm endosome-derived vesicles released from various cell types under both normal and pathopysiological conditions. They are derived from plasma membranes following endocytosis, where early endosomes are subsequently sorted into multivesicular bodies (MVBs). Inward budding of endosomal MVBs result in generation of intraluminal vesicles (ILVs). There are two distinct classes of MVBs including degradative MVBs which lead to lysosomal degradation and exocytic MVBs, which fuse with the plasma membrane and release ILVs from the cell in the form of exosomes. Exosomes are the only known secreted cellular vesicles derived from internal membranes [66]. Exosomes are shed by various myeloid and lymphoid cells including B and T lymphocytes, dendritic cells, microglia and macrophages, but also tumor cells of multiple lineages and neurons [67]. The molecular composition of exosomes is largely dependent on cell type, with exosomes from various origins containing both conserved proteins and cell type specific proteins. Most exosomes contain cytoplasmic proteins, members of endocytosis pathway proteins, and signal transduction proteins. In addition, heat shock proteins such as hsp70 and hsp90, and MHC class I molecules are commonly identified in exosomes [67]. For example, protein analysis of exosomes secreted from rat cortical neurons showed cytoskeleton, signaling, and membrane trafficking associated proteins including HSP70 family members [68]. Exosomes may contain multiple heat shock proteins under normal conditions, including hsp27, hsp60, hsp70, hsc70, and hsp90, with hsp70 (hsp72) levels being increased by heat stress [69] and intracellular bacterial infection [70]. It was reported that mycobacteria infection of macrophages enhances exosomal release and hsp70 content of exosomes [71]. Hsp70 has been localized to the surface of exosomes, placing it in a position to trigger innate immune signaling by interacting with cell receptors. 

Hsp70 release from viable virus-infected cells is a largely unexplored aspect of virus-host interaction. We are currently examining the release mechanism in MeV infected neuroblastoma cells, which may include exosomal release or microvesicular shedding. *In vitro* infections of human bronchial epithelia cells with RSV also cause hsp70 induction and extracellular release, and tracheal aspirates from RSV infected children showed increased levels of extracellular hsp70, although it was not determined whether the elevation of hsp70 was the result of active secretion or cell death [72]. It was also reported that the level of hsp70 in serum from infected patients was significantly increased relative to healthy control groups [73].

### 3.1. Extracellular hsp70 and Immunity

Extracellular hsp70, whether free or exosome-associated, is a potent stimulus for the induction of innate immunity and/or proinflammatory responses. Hsp70 is now included as one of the endogenous molecules in the list of damage-associated molecular patterns(DAMPs) that are recognized by Toll-like receptors (TLR) 2 and 4 [74]. TLRs were originally known as receptors recognizing pathogen-associated molecular patterns (PAMP), but it was subsequently recognized that TLRs also can respond to endogenous ligands including both HSP and non-HSP molecules [75,76]. Thirteen TLRs are found on the cell surface or within the cell. Exogenous hsp70 recognizes TLR2 and TLR4 to induce transcription of proinflammatory cytokines through MyD88 and NF-κB in human monocytes [77]. Hsp70-mediated production of proinflammatory cytokines was also demonstrated in microglia cells and macrophages isolated from mouse and rat, primarily through TLR4 [78,79]. *In vivo*, expression levels of TLR2, TLR4, MyD88 and hsp70 was measured in a traumatic brain injury model of rats using immunohistochemistry. Traumatic injury resulted in high level expression of MyD88, TLR4, and hsp70 in macrophages/microglia and astrocytes. Interestingly, hsp70 was expressed not only in macrophage/microglia and astrocytes but also neurons, such that injury of any one cell type (glia or neurons) could release hsp70 and thus result in TLR4 signaling [80]. Activation of TLR4 also triggers signal transduction for the induction of type I interferon by recruiting TRAM and TRIF, which leads to the activation of IRF-3 and NF-κB [79,81]. The potential for hsp70 to induce type I IFN has not however been investigated to date. In addition to triggering innate immune responses, released hsp70 may also have potential to stimulate adaptive immunity by binding antigenic peptides and cross-presenting these peptides to antigen presenting cells [82]. Here, hsp70 released from infected cells may be bound to viral peptides from the cell origin. The hsp70-peptide complex can then bind CD91 on antigen presenting cells for subsequent cross-presentation of the peptide on MHC I [83,84]. For example, hsp70 bound to specific cytotoxic T lymphocyte (CTL) epitopes of lymphocytic choriomeningitis virus can elicit virus-specific protective immune responses that are CTL mediated [85]. Immune stimulatory effects are a feature of multiple HSP family members, although hsp70 plays a key role. Heat shocked tumor cells release multiple HSPs including hsp60, hsp70 (hsc70 and hsp70), and hsp90 (hsp90 and grp94) but only hsp70 containing supernatants have potential to stimulate significant antitumor immune responses [86].

Given the potential of hsp70 to stimulate innate immune responses, it is considered an alarm signal to neighboring cells in various kinds of pathophysiological environments including surgical stress, physical stress, ischemia, inflammation, and hyperthermia [87,88,89,90,91,92]. Release of hsp70 into cerebrospinal fluid (CSF) of the central nervous system (CNS) is an emerging field of research. Increased hsp70 levels in CSF have been reported in association with the inflammatory response to ischemia-reperfusion injury in spinal cords of humans and dogs [92,93]. Data from the ischemic-reperfusion injury model in dogs supports secretory release from within the CNS since hsp70 levels in serum was unaffected [92]. We have also observed increased levels of hsp70 in cerebrospinal fluid (and not serum) in steroid-responsive meningoarteritis in dogs [91], again supporting the potential role of extracellular hsp70 as a mediator of primary CNS inflammatory responses. Release of extracellular hsp70 in response to viral infection of the CNS has not been reported, and in no instance of CNS inflammatory disease has the form of hsp70 release been characterized. The form of extracellular hsp70 (*i.e.*, free or exosomes associated) can significantly effect the potency for immune stimulation. For example, level of TNF-α induction in macrophages was 250-fold higher with membrane associated hsp70 compared to the same amount of hsp70 in solution [94]. The mechanism is thought to reflect the concentration of membrane-associated hsp70 and possible stabilization of an orientation that is optimal for TLR interaction. 

### 3.2. Hsp70 and Antiviral Immunity

Relevance of hsp70 to immune responses to viral infection has been vastly understudied, and largely restricted to examining how viruses may influence antitumor immunity. The latter is based upon the idea that extracellular hsp70, released by virus infection, can cross-present tumor-specific antigen to induce an effective antitumor immune response, or provide an adjuvant effect by simply activating innate immune responses [95]. Hsp70 has been shown to stimulate tumor specific T cells responses in a TLR2 and TLR4 dependent manner [96]. Virolytic tumor therapy is in part of an attempt to release heat shock proteins in order to stimulate a protective immune response, and hsp70 is released by tumor cells infected with parvovirus H1 to enhance anti-tumor immunity [65]. Adjuvant effects of hsp70 were demonstrated when examining immunogenicity of VSV-expressed cDNAs from a human melanoma library, with adjuvant effects associated with VSV induction of hsp70 expression [97].

Surprisingly little investigation has been focused upon the potential of hsp70 to influence immune responses to viral infection, outside of a small number of vaccine studies. Coexpression of Japanese encephalitis virus or HSV-1 structural protein with hsp70 is protective against subsequent lethal viral challenge in mice, where hsp70 expression is correlated to a stronger cellular immune response [98,99,100]. The extracellular release of hsp70 observed in bronchoalveolar lavage fluids of RSV infected children [51] is correlated evidence of leukocyte activation through TLR4 [72], however studies that directly examine the contribution of hsp70 to antiviral immunity and the impact upon viral virulence are lacking.

## 4. Hsp70-Mediated Host Protection in the Mouse Model of MeV Brain Infection

Our group has studied the effects of enhanced hsp70 expression on viral neurovirulence using the mouse model of brain infection. Mice are a good system to study the effects of increased hsp70 levels since the baseline in normal mice is low; mice do not constitutively express hsp70 in neurons (unlike other mammals) and they lack a febrile response following viral infection [58,101]. We can increase hsp70 expression levels by transient hyperthermic treatment or by selective transgenic expression. An additional benefit of the mouse system is that the hsp70 responsiveness of MeV is well characterized in mouse neuroblastoma cells [43] and neurons are a primary target of infection in the mouse model of MeV infection in brain. Intracranial injection of Edmonston MeV to neonatal mice exploits both mouse age and strain-dependent susceptibilities to MeV infection [60,102,103,104,105,106], eliminating the need for transgenic expression of the human membrane cofactor protein (CD46) as an ancillary viral receptor or use of mouse brain adapted strains of MeV in order to promote brain infection [107,108,109,110]. In addition, the high incidence of stable persistent infection and low incidence of mortality are helpful to study the hsp70-mediated effects that may either increase virulence or clearance. Mouse strains show differences in susceptibility to MeV infection that is associated with their H-2 haplotype, major compatibility complex(MHC) class I, II and III loci that determine the efficacy of immune responses to MeV following intracranial challenge [111,112]. The H-2 dependent basis for susceptibility has been related to differential IFN-γ production by MeV specific CD4^+^ T cells. IFN-γ production is enhanced in resistant mice strain carrying the H-2^d^ allele and diminished in susceptible mice strain carrying the H-2^b^ allele [113]. The different responses may reflect the activity of immune regulatory genes inducing cytokine expression, since *in vitro* and *in vivo* stimulation of lymphocytes from H-2^b^ mice showed decreased levels of Th1 cytokines resulting in lower amount of IFN-γ production relative to H-2^d^ mice [114]. 

Protective immunity in the mouse begins with innate immune responses of uninfected brain macrophages (microglia) to signals from infected neurons [115]. The identity of the signals has not been previously resolved. The key innate responses include production of type 1 interferon, which is predominantly IFN-β in the brain, and cross-presentation of viral antigen on MHC I [116]. MeV infection does not induce IFN-β in neurons [117,118,119], which may reflect a more general restriction of type 1 IFN production in this cell type, although neurons do maintain the capacity to respond to IFN-β by producing antiviral IFN stimulated gene products [117]. MeV also does not induce significant levels of MHC I on neurons, the induction being primarily observed on glia [118,120]. As such, virus-specific T cells primed in the periphery, respond to viral antigen that is cross-presented by activated microglia. These stimulated T cells produce IFN-γ that mediates non-cytolytic viral clearance [115]. The fundamental importance of IFN-β in this process extends beyond the antiviral state induced in neurons to include stimulation of microglial activation [121,122] and stimulation of virus-specific T cells to produce IFN-γ [123,124]. These combined effects make IFN-β expression a key determinant of permissiveness to (or protection against) viral neurovirulence. 

Initial studies on the effects of hsp70 on the outcome of MeV brain infection used transient hyperthermic treatment to induce hsp70 in neurons and glia of neonatal Balb/c (H-2^d^) mice [58]. Infection followed the hyperthermic treatment. Treated mice showed decreased MeV cytopathic effect, viral burden and increased clearance that was correlated to increased number of virus-specific lymphocytes in spleen relative to control mice. These data suggest that hyperthermic pre-conditioning, with the transient elevation in hsp70 levels, enhance the development of cell-mediated antiviral immunity. Unresolved is the influence of other heat induced stress proteins, or the different roles that heat shock proteins may be playing in virus infected cells relative to the uninfected brain macrophages that are key to innate and adaptive immune responses.

A transgenic model was used to establish the role of virus-hsp70 interaction in the infected neuron to influence innate and adaptive immune responses in uninfected brain macrophages and lymphocytes [61,125]. Transgenic mice that selectively overexpress hsp70 in neurons were created on an H-2^d^ background. The expression construct was driven by the neuron specific enolase promoter, and the neuron specific expression pattern was confirmed by immunohistochemistry. The hsp70 TG H-2^d^ mice showed complete protection against Edmonston MeV neurovirulence, compared to 35% mortality in NT controls. Survival analysis supported the statistical significance of the infection outcomes and showed an inverse correlation between survival time and brain viral RNA burden. Included in this study was a genetically engineered Edmonston MeV variant lacking a transcriptional response to hsp70. A single amino acid substitution in the C-terminus of the N protein (N522D) disrupts one of two hsp70 interacting sites, resulting in a selective disruption of hsp70-mediated stimulation of transcription without affecting other aspects of basal or hsp70-dependent replication [43]. Infection of TG mice with the N522D virus resulted in a level of mortality that was not significantly different from that observed in NT mice. Results thus show that a viral transcriptional response to hsp70 is essential to the complete host protection mediated by hsp70.

In order to study the basis for hsp70-mediated host protection, mice infected with MeV were depleted of CD4^+^ and/or CD8^+^ T cells via intraperitoneal injection of CD4 or CD8-specific monoclonal antibodies. Mortality was significantly increased from 35% to 94% in NT mice in which CD4^+^ T cells were depleted, but TG mice in which CD4^+^ T cells were depleted showed only 41% mortality. TG mice lacking both CD4^+^ and CD8^+^ T cells still showed a 27% survival rate. This result shows that hsp70 confers a significant enhancement of innate immunity against MeV-induced mortality. Infected brains were then analyzed at 5 days post infection (d p.i.) by real time RT-PCR of total brain RNA in order to gain insight as to the basis for hsp70-mediated innate immunity. Significant infiltrations of lymphocytes are not observed at 5 d p.i. based upon evaluation of tissue sections and low levels of IFN-γ transcripts in brain. Both lymphocytic infiltrates and IFN-γ was significantly increased at 10 d p.i., indicating the onset of adaptive T cell immune responses. The RT-PCR analysis showed enhanced activation of macrophages in TG relative to NT mice at 5 d p.i. based upon levels of CD68 and MHC II transcripts. We also showed greater levels of TLR2 and TLR4 transcripts in infected TG relative to NT mice.

**Figure 1:**
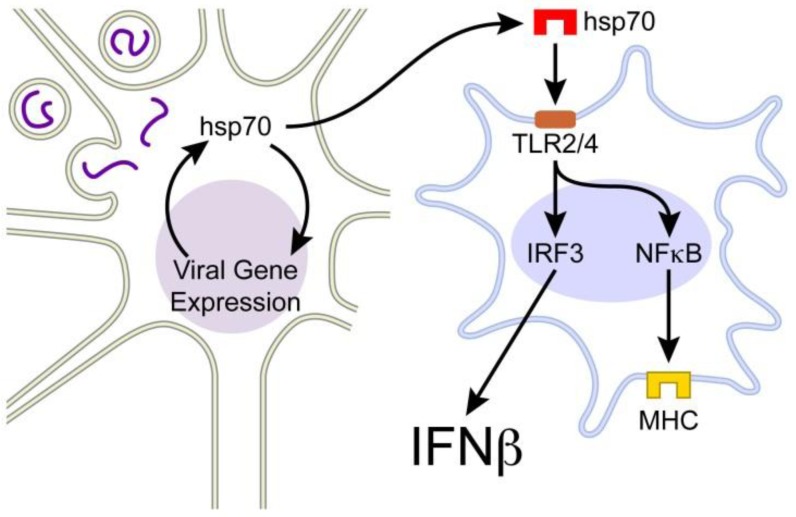
A novel axis of innate antiviral immunity, in which hsp70 is released from virus infected cells (neuron, left) to stimulate innate immune responses of macrophages (microglia, right). Virus core particles (purple nucleocapsids) in the neuron mediate gene expression that is stimulated by hsp70 and results in further hsp70 induction, setting into motion a positive feedback loop. The extracellular hsp70 is ligand for TLR2 and TLR4, activating signal transduction pathways that drive type 1 IFN expression (IFN-β in brain) and expression of antigen presenting complexes (MHC). IFN-β expression by brain macrophages is key to immunity against virus infected neurons.

Results from our laboratory and data in the literature combine support a novel axis of innate antiviral immunity that is driven by hsp70 (Figure 1). In this model, MeV-hsp70 interaction in neurons results in the extracellular release of hsp70. The level of release reflects the intracellular concentration of hsp70 and the magnitude of virus gene expression. The ability of hsp70 to stimulate virus gene expression and for virus gene expression to induce hsp70 thus represents a positive feedback loop driving hsp70 extracellular release. The extracellular hsp70 acts as a damage associated molecular pattern to stimulate innate responses of brain macrophages, particularly the expression of IFN-β. The IFN-β would create an antiviral state in infected neurons, would drive further brain macrophage activation and the cross-presentation of viral antigen derived from infected neurons. Virus specific T cells, encountering viral antigen expressed on activated brain macrophages, would secrete IFN-γ, resulting in viral clearance. IFN-β would be key to this aspect of the adaptive immune response as well. In this model, the extracellular hsp70 acts as a key danger signal, released by infected neurons, to stimulate host protective responses of brain macrophages. The relationship explains how the hsp70-mediated stimulation of virus gene expression is host protective, and not as paradoxical as it may seem. The relationship also provides a mechanistic basis for the host protective effects of fever, given that febrile temperatures are a potent inducer of hsp70 in brain. 

## 5. Future Directions

Ongoing studies using the MeV model of brain infection are designed to validate the model for hsp70-dependent innate immunity. Unpublished studies detail transcriptome analysis in brain RNA harvested from TG and NT mice at 5 d p.i. The top three canonical pathways that distinguish infected TG from infected NT mice are macrophage maturation, IFN induction and antiviral immunity, and antigen presentation. Results support a greater type I IFN response in TG relative to NT infected mice as the main differences linked to survival. Activation of brain macrophage and antigen presentation is supported by immunochemistry analysis of MHC II, and activation of type I IFN is confirmed by real time RT-PCR. Use of mice lacking a functional type I IFN receptor (IFNAR^−/−^) shows the critical role of type I IFN in hsp70-mediated protection. Cell culture studies confirm secretory release of hsp70 from MeV infected neurons and the ability of extracellular hsp70 to serve as a potent stimulus for the production of IFN-β in uninfected microglia.

The mechanism of innate immune activation by hsp70 suggests a broad virological and host relevance. Hsp70 stimulates gene expression of viruses in multiple families, fever is a frequent accompaniment of virus infection, ensuring elevated levels of hsp70 in virus infected cells, and both lytic and non-cytolytic infections can induce extracellular hsp70 release. As a TLR ligand capable of activating macrophages and inducing type 1 IFN, hsp70 can mediate induction of innate immune responses in a far greater sphere of uninfected cells than would be achieved by host responses to a pathogen associated molecular pattern. Work in progress will show this more broad relevance to rhabdoviruses, a family of negative strand RNA viruses that include rabies. Using vesicular stomatitis virus, we have shown that lytic infection of neurons results in high level release of hsp70, and that mice are protected against neurovirulence following a natural route of infection (*i.e.*, intranasal inoculation). Importantly, the protection is observed in weanling mice, proof that the hsp70 protective mechanism is not age-dependent. Although our initial focus has been the central nervous system, the axis of hsp70-mediated innate immunity should also apply to any organ system, reflecting the ubiquitous nature of hsp70 and TLR2/4 expression. Observations with RSV infected children support this contention.

## 6. Conclusion

*In vitro* studies showing that hsp70 stimulate virus gene expression has presented a paradox when considering the phylogenetic conservation of the febrile response to microbial infections. Fever is a potent inducer of hsp70 in all tissue and the conservation of this response suggests a host protective role. The apparent paradox can be reconciled by considering the potential of the increase in virus gene expression to stimulate innate immunity, particularly the expression of type 1 interferons. Historically, focus has been placed upon pathogen associated molecular patterns as the stimulus. The current review highlights the novel role of hsp70 as a damage-associated molecular pattern, released from infected cells and serving as a potent inducer of type 1 interferon. The relative importance of this novel axis of innate immunity is defined by the ubiquitous nature of hsp70 expression, the abundance within virus infected cells, and the apparent dependence of many viruses on hsp70 to support virus replication. The virus-hsp70 relationship is inescapable.
